# Gaps and paths forward in cancer pharmacology and translational research

**DOI:** 10.3389/fphar.2026.1779049

**Published:** 2026-04-10

**Authors:** Elizabeth A. Hughes, Lindsay L. Davenport, Christopher Zdyrski, Aleksandra Pawlak, Jonathan P. Mochel, Eugene F. Douglass

**Affiliations:** 1 Pharmaceutical and Biomedical Science, College of Pharmacy, UGA, Athens, GA, United States; 2 Precision One Health Initiative, Department of Pathology, College of Veterinary Medicine, Athens, GA, United States; 3 Precision One Health Initiative, Department of Physiology and Pharmacology, College of Veterinary Medicine, Athens, GA, United States; 4 Department of Pharmacology and Toxicology, Faculty of Veterinary Medicine, Wroclaw University of Environmental and Life Sciences, Wroclaw, Poland

**Keywords:** cancer drug discovery and development, drug efficacy, PK-PD (pharamacokinetic-pharmacodynamic) model, translational gap, translational medicine

## Abstract

As drug development costs continue to rise, there is a need to reframe how drug efficacy is evaluated in preclinical models to reduce the rate of false positives. The “valley of death” refers to the gap between bench research and clinical translation. In particular, oncology chemotherapies have the highest rate of drug failure compared to other drug classes. While there has been progress in overall cancer survival, some cancers and patient populations still have a poor prognosis. To bridge the gaps of drug failure and aid underserved patient populations, drug translation cannot be viewed as a purely linear process, but one in which continual refinement is used to create more efficacious drug candidates. Additionally, pharmacokinetic-pharmacodynamic modeling could prove instrumental in better understanding drug efficacy in cellular models and in evaluating clinical translation potential with greater accuracy. The path forward in clinical pharmacology is to view drug development and efficacy as a dynamic process rather than a purely linear fashion. This review discusses traditional pharmacodynamic and pharmacokinetic evaluation methods, as well as pharmacokinetic-pharmacodynamic models of tumor growth inhibition.

## Introduction

**FIGURE 1:**
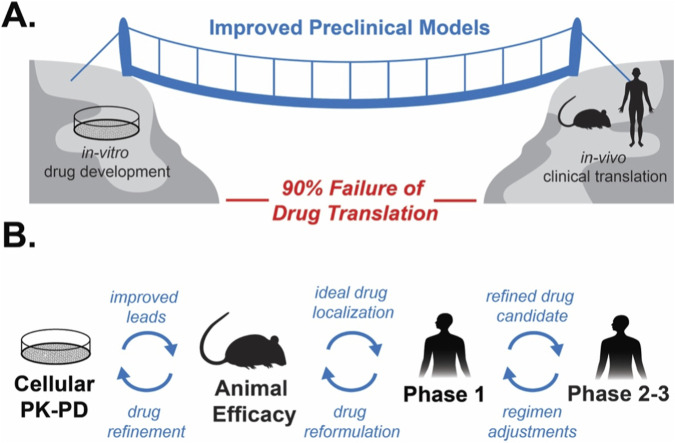
Translational gaps and utility of feedback loops. **(A)** Pharmacokinetic-Pharmacodynamic (PK-PD) modeling and improved *in vitro* modeling helping bridge the “valley of death” between *in vitro* drug development and clinical translation. **(B)** The utility of feedback loops and cellular PK-PD models to aid in drug development and drug translation.

Most drugs fail during the transition from preclinical research to clinical trials, resulting in a gap between laboratory research and patient care. The gap is often called the “valley of death,” where approximately 90% of drugs fail in clinical development following preclinical development, with 40%–50% failing due to a lack of clinical efficacy (Figure 1A) (Seyhan, 2019; Sun et al., 2022; Sorger, 2011). Even drugs that have optimal selectivity for target binding and are deemed ideal candidates can fail in clinical trials (Figure 1A). According to a 2011 NIH White Paper by the Quantitative and Systems Pharmacology Workshop, the cost of drug development—or, rather, its failure—is ultimately shouldered by patients, third-party payers, and the government (Sorger, 2011). It is projected that, under current attrition rates, drug development costs could reach $16B per drug by 2043 if innovations that reduce turnover in drug development and translational research are not implemented (Seyhan, 2019). With the rise of personalized medicine, identifying gaps from bench to bedside is imperative to provide patients with proper care and to develop novel therapeutics for underserved diseases. The term bench-to-bedside can refer to either bringing a drug to market for a novel target or disease, or matching patients to the right drug.

The gap between the laboratory research and clinical research is often referred to the “valley of death” (Seyhan, 2019; Sun et al., 2022; Hidalgo et al., 2014; Tentler et al., 2012; Wensink et al., 2021). There are a plethora of reasons making translational research difficult and expensive. The first being the lack of a single reason why drugs fail, meaning it varies case to case (Seyhan, 2019; Sun et al., 2022; Sorger, 2011). Additional reasons include off-target toxicity, similar efficacy to current drugs on the market, less-than-ideal pharmacokinetic (PK) parameters, a combination of multiple issues and/or lack of funding. There are two main areas where drug translation fails in human patients. Although first-in-human oncology studies are primarily designed to characterize safety and tolerability and define an appropriate dose and dosing schedule, Phase 1 trials increasingly integrate Pharmacokinetic-Pharmacodynamics (PK-PD) and biomarker strategies to evaluate target engagement and generate preliminary evidence of antitumor activity, informing maximum tolerated doses (MTD) and dose optimization for subsequent trials (Seyhan, 2019; Sun et al., 2022; Sorger, 2011; Li et al., 2024). Phase II oncology trials frequently fail, with reported attrition rates approaching 80% in some therapeutic areas (Seyhan, 2019; Sun et al., 2022; Aykan and Özatlı, 2020). Additionally, the main driver of clinical failure is no longer pharmacokinetics and drug exposure, but rather a lack of clinical benefit and insufficient efficacy, which account for approximately half of failures in Phase II clinical trials (Harrison, 2016). This lack of clinical efficacy can be traced to limitations in preclinical models and a purely linear view of drug development. Several mouse models are used to evaluate cancer progression and oncology drug response, with varying utility and clinical translation potential. One model type is engineered models, such as transgenic mice, which are genetically altered to mimic human cancers by carrying specific oncogenes or knocking out specific tumor suppressor genes (Zitvogel et al., 2016; Frese and Tuveson, 2007; Day et al., 2015). However, the most common are transplantable tumor models, also called xenograft mouse models, due to their low cost and reproducibility (Teicher, 2006; Zitvogel et al., 2016; Frese and Tuveson, 2007; Day et al., 2015). Patient-derived xenograft (PDX) models are more predictive of clinical response than cell line-derived xenograft (CDX) models (Sausville and Angelika, 2006; Johnson et al., 2001; Hidalgo et al., 2014). While efficacy in mouse studies often does not translate to the clinic due to differences in drug pharmacokinetics and immune system interactions, they remain the cornerstone for pharmacokinetic and toxicological evaluation and the design of Phase 1 dose-escalation studies in humans (Mak et al., n.d.; Lu et al., 2011; Sun et al., 2022; Teicher, 2006). This highlights the need for preclinical models beyond rodent work to improve clinical translation, including species such as dogs, which spontaneously develop diseases analogous to human disease and sometimes receive similar treatments (Schneider et al., 2018). Another limitation of Phase II trials is that the patient criteria are narrow, and even within those circumscribed cohorts, patient responses can vary, especially in oncology, due to the complexities of the tumor microenvironment and heterogeneity (Sun et al., 2022; Sausville and Angelika, 2006; Guillen et al., 2022; Narasimhan et al., 2020; Ganesh et al., 2019). Variations in patient genetics and drug resistance, both intrinsic and acquired, can also contribute to differences in patient response (Sorger, 2011; Moffat et al., 2017; Iorio et al., 2016). Additionally, in patients, it is difficult or even impossible to confirm target binding; only phenomenological data are evaluated to demonstrate a drug’s efficacy (Aykan and Özatlı, 2020; Eisenhauer et al., 2009; Schwaederle et al., 2015; 2016).

Another limitation is that drug efficacy is evaluated in the laboratory, which historically focuses on a pharmacodynamic (PD) and concentration-response paradigm, often emphasizing drug-target binding rather than disease response. The clinic, on the other hand, focuses on the change in the phenotypic response in relation to the pharmacokinetic parameters (PK) utilizing more of a PK-PD framework to evaluate drug efficacy. Differences in how drug efficacy is quantified further exacerbate the gap between the two disciplines. Additionally, many emerging treatment options, such as biologics (mRNA therapeutics, immune checkpoint inhibitors, and monoclonal and bispecific antibodies), drugs that alter protein-protein interactions or induce degradation (molecular glues and proteolysis-targeting chimeras), do not follow the traditional PD-Hill paradigm as efficacy is dependent on multiple targets and/or multiple cell types, requiring new *in vitro* methods such as co-culture, to evaluate drug efficacy (Shu et al., 2025; Spiridon et al., 2002; Douglass and Miller, 2025; Jenkins et al., 2018). Taken together, this highlights deviations from traditional *in vitro* cancer drug development, which has historically focused on improving drug potency in immortalized, homogeneous cancer cell lines that lack tumor heterogeneity and the tumor microenvironment. Alternatives to mouse models include human immortalized cancer cell lines, which, like CDX, have lower predictive power and high rates of false positives (Mak et al., n.d.; Sun et al., 2022; Carrera et al., 2018; Seyhan, 2019; Kurtz et al., 2017). More complex models, such as patient-derived organoids (PDOs) and 3D spheroids, offer greater translational potential (Ganesh et al., 2019; Guillen et al., 2022; Narasimhan et al., 2020; Wensink et al., 2021). Additional microphysical systems can offer more dynamic dosing and exposure control to better simulate drug distribution and elimination (Sánchez-de-Diego et al., 2025; Moshksayan et al., 2025; Gabriel et al., 2022). To improve drug translation, drug development cannot be considered a purely linear process (Figure 1B). We believe that feedback loops between PD-centric and PK studies are needed to bridge the gap from bench to bedside (Figure 1B) (Seyhan, 2019; Sorger, 2011; Fernald et al., 2024). While the *in vitro-to-in vivo* pipeline is somewhat established, evaluating cell models prior to animal studies and using previously published data further along in the translation process can help refine methods for future studies (Sausville and Angelika, 2006; Tosca et al., 2023). For example, if Phase I clinical trials show that the MTD or maximum plasma concentration (C_max_) is considerably lower than the IC_50_ (the concentration needed to induce 50% of the desired effect), studies can be conducted to either improve potency, the dosage form, or increase bioavailability (Figure 1B) (Sorger, 2011; Goldstein et al., 2021; Jansson-Löfmark et al., 2020; Kang et al., 2011).

The laboratory should potentially shift its focus to using both concentration- and time-based metrics more closely aligned with clinical endpoints, rather than primarily focusing on target binding. In addition, tissue-level PK and/or time-resolved drug exposure are commonly inaccessible or not incorporated into the laboratory, so there is a need for additional databases to identify gaps in clinical literature. Databases such as the Cancer Cell Line Encyclopedia (CCLE) and The Cancer Genome Atlas (TCGA) enable scientists to gain a deeper understanding of the impact of genomics and proteomics on drug response (Sorger, 2011; Barretina et al., 2012; Iorio et al., 2016). We believe that by curating and developing additional databases for animal pharmacokinetic and Phase I data, we can refine the laboratory’s understanding of drug exposure and drug response. We believe that integrating PK-PD principles into preclinical models is necessary but, by itself, insufficient for successful clinical translation. In addition to reframing the conceptual framework, to improve our bench-to-bedside paradigm, the field needs to incorporate more robust *in vitro* and *in vivo* models, as well as advanced *in silico* approaches for data integration, mechanistic inference, and prediction. This review aims to discuss the current paradigms of drug efficacy and translation, their limitations, and avenues for innovation to bridge the “valley of death.”

## Pharmacodynamics and common models

### What is PD?

Pharmacodynamics describes the relationship between drug concentration and biological effect, which, in the context of oncology, is typically evaluated as the ability to induce cytotoxic or cytostatic responses in cancer cells (Meibohm and Derenforf, 1997). This relationship has historically been formalized through classical receptor occupancy–based PD models, originating from Paul Ehrlich’s concept of selective drug–target interaction and later developed by A.J. Clark and E.J. Ariëns, which relate drug concentration, receptor engagement, and efficacy to downstream effect (Ing, H.R. 1943; Ariëns, 1983; Kaufmann, 2008). This is frequently quantified by the relationship between drug concentration and drug effect at steady-state concentration (Meibohm and Derenforf, 1997). Current drug discovery efforts are focused on concentration-response PD models. While these models are beneficial, they are typically used with a single time point, overlooking the dynamic nature of the drug response process. Many drug discovery groups have begun to incorporate time into their assay designs (Ceribelli et al., 2024; Duellman et al., 2015; Evans et al., 2019; Polanco et al., 2022). Preclinically, this is critical because a minimum drug exposure time is required to translate “target binding” into a downstream “therapeutic response” (Hafner et al., 2016; 2017; Tyner et al., 2013; Hsieh et al., 2017). This downstream signaling, along with the core concepts of PD-PK modeling, is consistent with the clinical observation from early-phase precision oncology trials showing that, even with biomarker-selected patient selection, matched targeted therapies achieve partial or complete responses, as defined by RECIST criteria, in only approximately 30% of patients in early-phase clinical trials (Schwaederle et al., 2015; 2016). Similarly, in a large-scale pharmacogenomic analysis across ∼1,000 cancer cell lines spanning multiple tissue origins and ∼1,000 drugs, baseline target mRNA expression explains less than 30% of the variance in drug sensitivity end-point metrics, such as IC_50_ or Area Under the Curve (AUC) (Iorio et al., 2016; Basu et al., 2013; Corsello et al., 2020). Taken together, target-binding (dose-dependent) only partially explains cellular and clinical responses, indicating a more complex model of drug response that could be explained by drug kinetics. Therefore, both drug concentration and exposure time contribute to clinical efficacy, yet most laboratory studies focus on the former (i.e., IC_50_) (Lombardo et al., 2018). Therefore, a more comprehensive understanding of drug efficacy, extending beyond traditional pharmacodynamics, and the necessity of more complex models to account for the various factors influencing drug response are warranted. While several models predict drug response across concentrations, the most common in cancer pharmacodynamics is the Hill equation (Goutelle et al., 2008; Gesztelyi et al., 2012). More recent manuscripts are adopting the Growth Rate Inhibition (GRI) model proposed by Hafner et al. (2016), Hafner et al. (2017).

## Dose potency (Hill)

### Dose-response background (clinical pros and cons)

**FIGURE 2:**
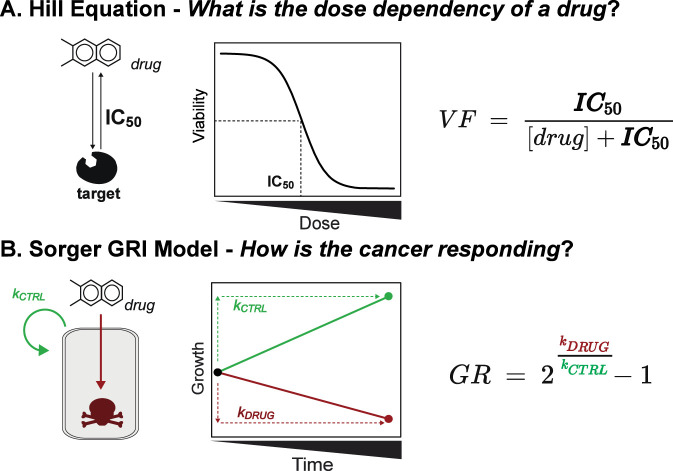
Metrics to determine cancer drug efficacy *in vitro*. **(A)** The Hill Equation is used to determine IC_50_, the concentration to induce 50% of the desired effect, in the case of cancer is based on the viability fraction, the percentage of viable cells. **(B)** Sorger’s GRI model uses Growth Rate (GR) to determine the overall trend of the cells at a concentration, whether they are proliferating (GR > 0), cytostatic (GR = 0), or dying (GR < 0). This is determined by comparing the rate of treated cells (k_DRUG_) to the rate of untreated cells (k_CTRL_).

In the early phases of current drug discovery, the goal is to optimize the concentration-dependent response by evaluating and optimizing drug potency, either by determining the maximum desired effect parameter (E_max_) or the concentration at which 50% of the desired effect is achieved (IC_50_) (Figure 2A) (Sorger, 2011). In oncology drug development, the goal of this approach is to prevent potential off-target effects, but this assumes that there is a set threshold for toxicity and efficacy across all drugs. In fact, most medicinal chemistry textbooks cite go/no-go potency thresholds for lead compounds with IC_50_ values below 1 µM (Gleeson et al., 2011). While these initial IC_50_ values can guide dose-escalation studies for animal and Phase 1 clinical trials (Huber and Mistry, 2024; Goldstein et al., 2021), some highly efficacious drugs, such as 5-fluorouracil and fluconazole, have both IC_50_ and C_max_ values far above 1 μM, with 5-fluorouracil being one of the most commonly indicated oncology treatments, and fluconazole being commonly used in antifungal therapy.

Additionally, it has been shown that the IC_50_ for the same drug-cell line pair can vary significantly between studies, depending on media conditions, passage number, and other experimental factors (Hafner et al., 2016; 2017). In addition to media conditions, a recent study conducted at the National Cancer Institute (NCI) across a comprehensive library of clinical oncology drugs found that the IC_50_ values reported were directly affected by the assay time point, thereby determining concentration dependence at standard time points of 48 and 72 h (Sorger, 2011; Evans et al., 2019). This observation of a minimum time scale required for potency is well understood in both the antimicrobial field and is increasingly recognized as necessary for emerging “catalytic” drugs such as bispecific antibodies, molecular glues, and proteolysis-targeting chimeras (Douglass and Miller, 2025; Kowalska-Krochmal and Dudek-Wicher, 2021). These studies highlight the dynamic nature of concentration-dependent efficacy metrics and show that standard dose-dependent metrics are typically static (single time point), so they may not reflect efficacious (IC_50_) or toxic (C_max_) thresholds on *in vivo* timescales. Preclinically, this is consistent with cell- and animal-based assays; a specific drug exposure time is required to translate “target binding” into a downstream “therapeutic response” (Hafner et al., 2016; 2017; Tyner et al., 2013; Hsieh et al., 2017). Implicitly, a single time-point fails to consider the minimum time necessary for a drug to induce its desired effect. The antimicrobial drug discovery field has adopted time- and dose-dependent metrics to match drugs to patients more effectively and to improve clinical translation by examining the time above the minimum inhibitory concentration (MIC), yet this approach is underutilized in the oncology field (Kowalska-Krochmal and Dudek-Wicher, 2021). Overall, *drug concentration* and *exposure time* contribute to *in vitro* and clinical efficacy, yet most *in vitro* cancer studies focus primarily on IC_50_ for fixed, specified time intervals (Lombardo et al., 2018).

### Time matters in the clinic

In the clinic, cancer treatment response is determined using the Response Evaluation Criteria in Solid Tumors (RECIST), which assesses treatment efficacy by quantifying changes in tumor size following treatment (Eisenhauer et al., 2009; Aykan and Özatlı, 2020). There are four post-treatment response categories according to RECIST. Complete response (CR) which is the disappearance of all target lesions, partial response (PR) is a decrease in tumor size by at least 30%, stable disease (SD) which is less than 30% shrinkage and less than a 20% increase, and finally progressive disease (PD) which is a >20% increase in tumor size (Eisenhauer et al., 2009). In clinical trials, drug efficacy is defined by the objective response rate (ORR), which measures the percentage of patients who achieve a partial or complete response after treatment (Aykan and Özatlı, 2020; Eisenhauer et al., 2009). These studies focus on how well a specific dose, as outlined in cell assays, affects treatment outcomes. Evaluation of disease progression (i.e., time-response) is commonly used in both animal models and antimicrobial research, but is underutilized in *in vitro* oncology research (Hafner et al., 2016; Kowalska-Krochmal and Dudek-Wicher, 2021). Recent publications advocated making a stronger comparison between cell-based assays and clinical metrics (Supplementary Figure S1), such as Sorger’s Growth Rate Models, which emphasize disease progression as the efficacy metric (Hafner et al., 2016; 2017; Evans et al., 2019; Fallahi-Sichani et al., 2013; Arbabi Moghadam et al., 2020). While traditional Hill concentration-response metrics can help identify whether a drug induces cytotoxicity, they do not always accurately reflect whether the model represents progressive disease, stable disease, or a partial or complete disease response (Hafner et al., 2016; 2017; Fallahi-Sichani et al., 2013). Limitations of the Hill equation include the assumption that potency and clinical efficacy are limited by the fact that concentration is not stable in both animal and human systems, and that drug efficacy can vary over time, depending on the drug’s pharmacokinetic behavior (Meibohm and Derenforf, 1997; Gesztelyi et al., 2012; Evans et al., 2019; Douglass, Jr et al., n.d.). The dynamic nature of drug concentration *in vivo* means that there is no single *in vitro* concentration analogous to the plasma concentration at any given time point (Lombardo et al., 2018). The exception might be continuous infusion regimens, but most assay time points are past the typical infusion time, which is typically less than 24 h (DeVita Jr et al., 2019). Additionally, even at a steady drug concentration *in vitro*, IC_50_ values vary between publications and time points (24, 48, 72 h). While the AUC accounts for the dynamic nature of concentrations observed *in vivo*, AUC and IC_50_ values are often reported as a single endpoint and a static *in vitro* potency metric (Evans et al., 2019; Benet and Zia-Amirhosseini, 1995). This difference in concentration over time (dynamic: *in vivo*; static: *in vitro*), along with the time-dependency of drug potency, shows that the traditional static end-point laboratory metric of *in vitro* potency is related to, but not the same as, *in vivo* efficacy (Jansson-Löfmark et al., 2020; Hafner et al., 2016; 2017; Huber and Mistry, 2024; Fallahi-Sichani et al., 2013; Johnson et al., 2001). As previously mentioned, in patient cancer treatment, drug efficacy is determined by the change in tumor size following treatment (Eisenhauer et al., 2009), so even if a point represents a strong responder at the IC_50_, that may not always be accurate.

### Hill equation

Laboratory drug efficacy is defined by the ligand-receptor theory, which has remained essentially unchanged over the past 100 years (Gesztelyi et al., 2012; Goutelle et al., 2008). Paul Ehrlich, the father of chemotherapy, first proposed in 1899 that drug efficacy was mediated by a single molecule binding to a single target (Kaufmann, 2008). In 1910, Archibald Hill observed the concentration-response behavior of oxygen binding to hemoglobin (Gesztelyi et al., 2012; Goutelle et al., 2008). Hill used this concentration-dependent behavior to derive the Hill equation (Figure 2A), making Elrlich’s theory quantitative (Gesztelyi et al., 2012; Goutelle et al., 2008; Kaufmann, 2008). From this equation, two of the most used metrics in pharmacodynamics are described, *K*_*d*_ and IC_50_ (Figure 2A). In the laboratory, drug optimization focuses on concentration and IC_50_ optimization, assuming that “best potency is equal to the best drug,” thereby optimizing for the therapeutic range, which is the concentration range where an effect can be seen (Liston and Davis, 2017), rather than efficacy, as illustrated in Figure 2B (Gleeson et al., 2011; Lombardino and Lowe, 2004). As mentioned above, pharmacodynamic models typically define lower and upper asymptotes (E_min_ and E_max_) that describe minimal and maximal observable effects across a concentration range (Figure 2A) (Gesztelyi et al., 2012; Goutelle et al., 2008). The traditional threshold metric, IC_50,_ is typically reported at a single time point, usually 24, 48, or 72 h, as in the NCI study (Hsieh et al., 2017; Evans et al., 2019).

### Sorger–growth rate inhibition

In 2016, Sorger and colleagues noted that not only does the IC_50_ vary across time points, but the cell proliferation rate is also an essential confounding factor in IC_50_ variations (Hafner et al., 2016; 2017; Fallahi-Sichani et al., 2013). Sorger proposes a new approach to understanding drug efficacy that considers cell proliferation, noting that the IC_50_ can vary across the literature for the same drug-cell line pair due to variations in media conditions and other factors that affect cell proliferation rates within the same studies (Hafner et al., 2016; 2017). Additionally, as previously described, in both clinical and preclinical *in vivo* cancer treatment, drug efficacy is assessed by changes in tumor size following treatment (Aykan and Özatlı, 2020; Eisenhauer et al., 2009). For this reason, both Sorger’s method and clinical practice rely on a baseline pre-treatment (Time 0) cancer-burden measurement to account for cell proliferation and differences in fundamental kinetic parameters with the aim of establishing a stronger correlation with clinical metrics for progressive disease, stable disease, and partial/complete responders (Hafner et al., 2016). Sorger et al. propose a new metric, Growth Rate (GR), to assess changes in cell density from the beginning of treatment and to determine whether the chemotherapy has no impact on cell growth, thereby creating a metric closer to the RECIST criteria for disease progression following treatment. GR determines if each drug has no inhibition on cell growth (GR > 0), slowed cell growth (GR = 0), or can induce a partial or complete inhibition of cell growth (GR < 0) (Supplementary Figure S1) (Hafner et al., 2016; 2017). The GR value is determined by the fraction of the change in growth of treated cells to that of untreated cells (Figure 2B). The benefit of this model is its ease of experimental implementation, as it requires only a single time point prior to treatment in addition to the traditional post-treatment time point. Additionally, the calculations are simple and do not require complex mathematical modeling. This metric is also more comparable to clinical response, as it can be used to identify which drugs have the most significant effect and to understand how the cancer responds to a drug (Supplementary Figure S1).

Growth rate, by contrast to Viability Fraction, is a comparative measure of how well a drug inhibits cancer growth relative to the cancer’s initial size (Hafner et al., 2016; 2017). This is implemented by taking two time points, one at the initial seeding density and one at the final endpoint, across a standard concentration gradient (Hafner et al., 2016; 2017). This allows us to assess the concentration potency of a drug and its ability to inhibit proliferation, where a minimum concentration is required to maintain tumor size/cell count, and a minimum concentration is required to decrease tumor size/cell count. This metric accounts for variation in proliferation across experiments, but due to the lack of sampling and temporal resolution, there is no observable time lag in the drug response. However, this constant monotonic model assumes immediate drug action, which is incompatible with observations in animal and cell-based assays, where different drugs require a minimum exposure time to induce the desired effect (Polanco et al., 2022; Gross et al., 2023). To understand the dynamic nature of drug behavior, a system that integrates pharmaockinetics and pharmacodynamics can potentially aid in improving drug selection and clinical translation.

## Pharmacokinetics and common parameters

### What is PK?

**FIGURE 3:**
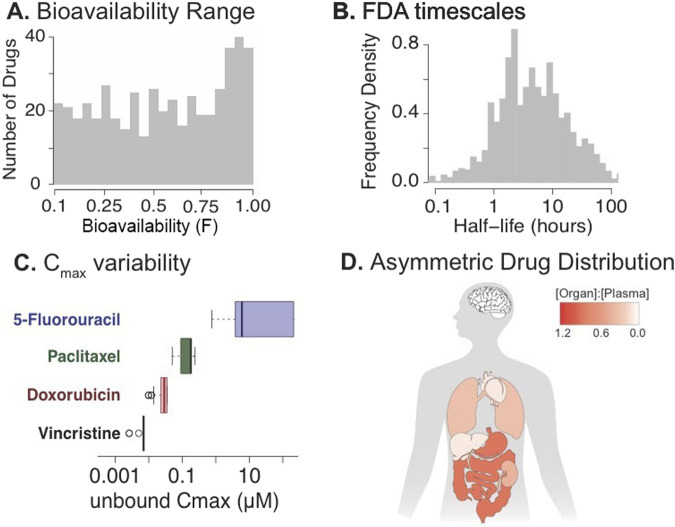
FDA metrics for oncology drugs. **(A)** The oral bioavailability range for FDA approved drugs (Lombardo et al., 2018). **(B)** The range of half-life in hours of FDA approved drugs (Lombardo et al., 2018). **(C)** The Maximum Plasma Concentration variability across four of the most commonly used oncology drugs where the dots represent data points outside of the interquartile range (Lombardo et al., 2018). **(D)** An example of asymmetric drug distribution across organ tissues (Hughes et al., 2023).

Pharmacokinetics describes the relationship between the body’s ability to alter the drug’s concentration and location following administration and is evaluated by the relationship of drug concentration over time (Meibohm and Derenforf, 1997; Benet and Zia-Amirhosseini, 1995; Sorger, 2011). A drug’s pharmacokinetic parameters determine whether a lead compound has poor or good drug-like properties and is ready for clinical trials. In the 1990s, poor-drug like properties and PK parameters were a large contributor to drug failure, but classical ADME/developmental failures only account for 10%–15% of drug failure today (Sun et al., 2022; Benet and Zia-Amirhosseini, 1995). In 2001, Pfizer’s medicinal chemist Christopher Lipinski developed the “rule of five”, which has contributed to our understanding of drug-like properties (Sun et al., 2022; Gleeson et al., 2011; Hughes et al., 2023; Lipinski et al., 2001). This rule is based on the observation that most orally bioavailable drugs have a molecular weight less than 500, a lipophilicity of less than 5, fewer than 5 hydrogen-bond donors, and less than 10 hydrogen-bond acceptors (Lipinski et al., 2001). While many drugs fall outside of Lipinski’s rules, these considerations, along with more rigorous testing of drug-like properties in cell, animal, and computational models, have helped reduce the risk of drug failure due to PK considerations. Pharmacokinetics is separated into four main areas of study: Absorption, Metabolism, Excretion and Distribution. The most commonly studied and understood sections are Absorption, Excretion, and Metabolism because they are easy to quantify (Benet and Zia-Amirhosseini, 1995). Absorption is the rate at which a drug enters the systemic circulation, determined by the rate constant of absorption (ka) and the oral bioavailability (F). Since oral administration is the preferred method for most healthy patients, a general rule of thumb of greater than 30%–50% oral bioavailability is considered optimal for most lead compounds (Figure 3A) (Sun et al., 2022; Davies et al., 2020). These parameters help determine the dose required for each patient based on the desired plasma concentration. Excretion and Metabolism are often linked together as the body’s detoxification method (Benet and Zia-Amirhosseini, 1995). Together, these mechanisms determine the drug’s clearance rate and half-life. Hepatic (metabolism) and renal (excretion) are the two main routes for drug elimination (Benet and Zia-Amirhosseini, 1995). Renal clearance is driven by glomerular filtration, as well as by passive and active transport in the kidneys. In contrast, hepatic clearance is driven by the breakdown of the drug through metabolic enzymes (i.e., the cytochrome P450 family) (Benet and Zia-Amirhosseini, 1995; Kivisto et al., 1995). Half-lives greater than 4 h are generally cited as the threshold for strong drug candidates, but similar to Lipinski’s rules, many drugs fall below this range depending on the disease, mechanism, therapeutic range, administration route, and/or other factors (Figure 3B) (Sun et al., 2022; Davies et al., 2020; Lombardo et al., 2018). These parameters allow us to determine the drug dose and dosing regimen based on the drug’s therapeutic range (DeVita Jr et al., 2019). Historically, in oncology, the C_max_ is often equivalent to the MTD and much higher than the concentration at the desired site of action, but many novel drugs, such as antibody-drug conjugates and immunotherapies, decouple C_max_ from toxicity (Figure 3C) (Rizk et al., 2017; Wolska-Washer and Robak, 2019; Le Louedec et al., 2020).

### Drug distribution

The least well-understood and studied area of pharmacokinetics is drug distribution (Benet and Zia-Amirhosseini, 1995; Eichler and Müller, 1998). Drug distribution is roughly quantified by the volume of distribution (V), a proportionality constant that captures the amount of drug distributed throughout the body at a given time point (Toutain and Bousquet-Mélou, 2004). Often, volume of distribution is approximated by the central volume of distribution (V_c_), which is estimated by dividing the dose by the extrapolated plasma concentration at time 0 (C_0_) (Benet and Zia-Amirhosseini, 1995; Toutain and Bousquet-Mélou, 2004). Most drugs have a volume of distribution significantly larger than the total volume of the human body because of a drug’s absorption into the tissues (Lombardo et al., 2018; Benet and Zia-Amirhosseini, 1995). A common assumption is that drugs are uniformly distributed throughout the body, but the most well-established exception is that most drugs have poor brain penetration (Figure 3D). Often, drug distribution is not driven by simple diffusion through tissues because of structural and functional barriers within the endothelium. Another contributing factor to drug distribution and the time it takes for a drug to distribute is drug transporters (Hughes et al., 2023; The International Transporter Consortium et al., 2010; Zhang et al., 2019; Bendayan et al., 2002; Scherrmann, 2002). There are two major classes of drug transporters: ATP-Binding Cassette and Solute Carriers (Giacomini et al., 2010). Of the ATP-binding cassettes, ABCB1 and ABCG2 are the most studied in their role in drug distribution (Giacomini et al., 2010; Dolghih and Jacobson, 2013; Aller et al., 2009). The Organic Anion Transporters are the most common subfamily within the Solute Carriers (Giacomini et al., 2010). Variation in the expression of these transporters and differences in structural barriers create an asymmetric distribution between tissue compartments (Figure 3D) (Hughes et al., 2023). While potential drug candidates may have excellent drug-like properties, if they have sub-therapeutic distribution to the disease tissue or tumor, poor efficacy may be observed in the clinic and not *in vitro* (Sun et al., 2022; Rizk et al., 2017; Gao et al., 2022). The assumption that all drugs are equally privileged to the unbound drug in plasma is misleading for many compounds that exhibit asymmetric tissue distribution (Figure 3D) (Sun et al., 2022; Hughes et al., 2023; Zhang et al., 2019). Measuring this drug tissue distribution can be costly and invasive, even in animal studies (Eichler and Müller, 1998). Tools such as magnetic resonance spectroscopy, single-photon emission computed tomography, and microdialysis can help researchers study the drug’s distribution over time (Eichler and Müller, 1998; Saleem et al., 2000; Dunphy and Nagavarakishore, 2020). Advances in radiotracer methods have enabled less invasive monitoring of drug distribution and localization within the tumor (Saleem et al., 2000; Morgenroth et al., 2019; Dunphy and Nagavarakishore, 2020). Cellular models, such as Caco-2 and MDCK, can also help predict drug distribution across tissue (Eichler and Müller, 1998; Siissalo, 2009; Sampson et al., 2015). A literature curation of 73 drugs across 23 tissues has shown that drug distribution is often not uniform in animal studies (Hughes et al., 2023). Typically, these studies have only one time-point, which does not provide an accurate assessment of changes in concentration over time. This is important because there are two observed processes of drug distribution: rapid *versus* delayed, which can lead to a delay or lag in the desired effect (Meibohm and Derenforf, 1997; Zhang et al., 2019). An example of delayed distribution is the time it takes Ibuprofen to reach its peak concentration relative to the onset of the desired effect (pain relief or fever reduction) (Rizk et al., 2017; Eichler and Müller, 1998). As mentioned, V is often reported as V_c_; this assumption is limited as most drugs demonstrate a slower distribution phase, so some of the decrease in drug concentration over time is related to drug partitioning rather than elimination (Toutain and Bousquet-Mélou, 2004). Differences in distribution time scales, along with drug signaling cascades, are often summarized in distinct compartments in PK-PD models (Meibohm and Derenforf, 1997). Additionally, because drug concentrations under non-steady-state conditions vary over time, altering both plasma exposure and drug levels at the site of action, drug effects are often assumed to be directly linked to instantaneous plasma concentration (Meibohm and Derenforf, 1997; Rizk et al., 2017; Zhang et al., 2019). A critical implication is that many reported barrier ratios do not represent equilibrium, as they are frequently calculated from single time-point measurements in barrier tissues with slow equilibration, leaving uncertainty about whether steady state has been reached. The assumption is that plasma concentration is correlated with the concentration at the site of action. If the concentration in the diseased tissue is below the therapeutic range, the drug may fail due to poor penetration into the disease site, which is critically important for drugs with low therapeutic margins and major off-target toxicities (Meibohm and Derenforf, 1997; Rizk et al., 2017; Eichler and Müller, 1998; Zhang et al., 2019; Gao et al., 2022). These types of failures in clinical translation could have been potentially identified earlier through tissue localization studies or by further developing drug localization to enhance clinical efficacy through improved *in vitro* drug distribution studies, newer *in vitro* methods simulating drug distribution through microfluidics, and utilizing nuclear drug tracing in early clinical studies.

## Pharmacokinetic-pharmacodynamic tumor growth inhibition

### What is PK-PD?

**FIGURE 4:**
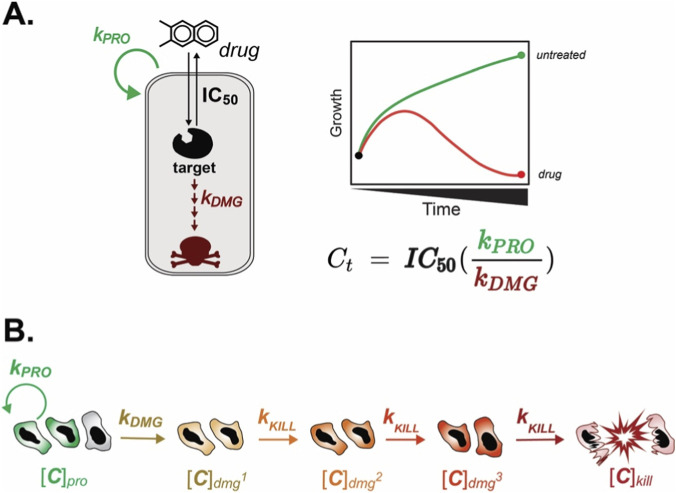
Simeoni tumor growth models in cancer pharmacology. **(A)** The Simeoni Tumor Growth Inhibition (TGI) model was developed in mouse xenograft data to determine the kinetics of drug efficacy on tumor growth. This is used to determine the Concentration Threshold (C_t_) which is the steady state plasma concentration needed to induce 100% tumor eradication. This is determined by multiplying the IC_50_ by the rate of proliferating cells (*k*_*PRO*_) over the rate of cells going through the damaging pathway (*k*_*DMG*_). **(B)** The progression of cell states and drug kinetics separated over time creating a lag between drug administration and cytotoxicity. The different parameters are determined through a series of differential equations (Simeoni et al., 2004; Rocchetti et al., 2007).

Drug response is a complex process that involves both the concentration-effect relationship and the concentration-time relationship within the body (Figure 4) (Sorger, 2011; Meibohm and Derenforf, 1997). Pharmacokinetic-pharmacodynamic is the study of how the drug’s desired effect changes over time in response to a particular drug concentration(s) (Sorger, 2011; Meibohm and Derenforf, 1997). When both the drug’s pharmacokinetic and pharmacodynamic properties are characterized, PK–PD models offer a structured framework for estimating maximal effects and duration of action; however, these estimates are inherently conditional and may be limited by biological heterogeneity, resistance mechanisms, and uncertainty in drug exposure (Meibohm and Derenforf, 1997; Simeoni et al., 2004). In early pharmacokinetic models, it was assumed that plasma concentration did not affect the drug’s pharmacological effect (Meibohm and Derenforf, 1997). Until the 1980s, when Sheiner et al. proposed the first compartment model to account for the lag between plasma concentration and the desired effect (either due to drug distribution or to delays in signaling cascades) (Nicholas and Sheiner, 1982; Sheiner et al., 1979). One limitation is that, under most non-steady-state concentrations, the drug concentration and its effect must be thoroughly defined to model the concentration-effect relationship (Benet and Zia-Amirhosseini, 1995; Nicholas and Sheiner, 1982). Another is that models alone cannot explain all variations and are unable to parse out whether poor drug response is due to a lack of drug accumulation or drug resistance (Sun et al., 2022; Sorger, 2011; Meibohm and Derenforf, 1997). The Quantitative Systems Pharmacology White Paper states that *“even at the level of cellular pharmacodynamics, understanding drug action requires integration of effects within the myriad interconnecting multi-pathway networks of signaling, metabolism, and gene expression; moreover, operation of these pathways involves molecular communication across cell boundaries into the microenvironment”* (Sorger, 2011). While pharmacology is a complex process, PK-PD models allow for the understanding of drug dynamics and lag effects in cancer tumor growth.

## Tumor growth inhibition models

### Untreated tumor growth

To understand how therapeutic agents can inhibit tumor growth, the kinetics of untreated tumor growth (rate constant *k_pro_* in Figure 4) must first be understood. The traditional model of cell growth is exponential, but this concept applies only to the early phases of tumor growth (Gerlee, 2013; V. G. Vaidya and Alexandro, 1982). Historical studies of xenograft models have demonstrated that tumors comprise two distinct cell populations: growing and non-growing cells (Koch et al., 2009; Simeoni et al., 2004; Benzekry et al., 2014). Modern tumor biology shows that these cell populations in tumors are continuous, reversible phenotypic states rather than discrete compartments (Whiting et al., 2024; Kholodenko et al., 2023). In addition to the different populations, initial tumor growth occurs in a nutrient-dense environment (Koch et al., 2009; Benzekry et al., 2014). In contrast, later disease progression occurs in an environment with fewer readily available nutrients, and the presence of a hypoxic core slows tumor growth kinetics (Koch et al., 2009; Benzekry et al., 2014). These observations resulted in a simplified two-phase tumor growth model, characterized by an initial exponential growth phase followed by a slower linear phase (Figure 4A, green curve) (Benzekry et al., 2014). The Gompertz equation was first applied to tumor growth in 1934 by Albert Casey to describe its biphasic, two-state growth pattern (Rygaard and Spang-Thomsen, 1997; V. G. Vaidya and Alexandro, 1982; Arbabi Moghadam et al., 2020; Gerlee, 2013). This model has historically been applied to both treated and untreated samples to evaluate changes in growth rate to assess drug efficacy in xenograft models and, more recently, in cell-based assays (Rygaard and Spang-Thomsen, 1997; Arbabi Moghadam et al., 2020). An additional model of tumor growth, the Bertalanffy model, was developed in the 1960s. These two growth rate models have laid the foundation for applying PK-PD modeling to traditional tumor growth models (Gerlee, 2013; V. G. Vaidya and Alexandro, 1982). While this concept has laid the foundation for most PK-PD tumor growth models, there is substantial variation in tumor growth patterns that often cannot be simplified to a two-phase growth pattern due to the highly heterogeneous environment within cancer (Schosserer et al., 2017; Morton et al., 2011; J. S. Vaidya, 2007). Two popular PK-PD Tumor Growth Inhibition (TGI) models for transitioning between these phases include Simeoni’s model, which assumes tumor growth abruptly switches from exponential to linear growth once a threshold is reached (Simeoni et al., 2004), and Koch’s closely related model, which suggests that tumor growth slows continuously throughout the process (Koch et al., 2009).

### Killing theories and drug kinetics

In oncology, the lag between drug administration and drug response can appear days to weeks post-treatment. In the 1970s, two main theories were developed to describe how drug treatment inhibits tumor growth: the Skipper-Schabel-Wilcox Hypothesis (“Log Kill”) and the Norton-Simon Hypothesis (West and Newton, 2017). The Log Kill Hypothesis states that chemotherapies kill a constant fraction of tumor cells, regardless of their size, and that each treatment decreases the tumor by the same fraction (Skipper, n.d.; Howard, 1965; Howard et al., n.d.). The Norton-Simon hypothesis states that the rate of drug damage is proportional to the rate of tumor growth at the time of treatment, laying the foundation for the idea that “faster growing cancers are more chemosensitive” because most conventional chemotherapies target actively, rapidly dividing cells (Norton and Simon, 1977). These two conceptual frameworks laid the foundation for current oncology practice in the development of dosing regimens. The Log-Kill hypothesis emphasizes the need for multiple cycles of chemotherapy (Skipper, n.d.; Howard, 1965), and the Norton-Simon model proposes the need for dose-dense treatment in earlier stages of disease (Norton and Simon, 1977; López et al., 2019). Both of these frameworks influenced the assumptions underlying the kinetics of drug response, which is modeled in TGI models. As mentioned earlier, there is a delay between drug administration and drug response (Meibohm and Derenforf, 1997). In TGI models, the delay can be explained by two distinct concepts: the Signal Distribution model and the Cell Distribution model. These ideas can be separated into whether the drug’s effect is direct (Cell Distribution model) or indirect (Signal Distribution model). In the Cell Distribution model, a drug can induce direct damage by altering the cell state from a proliferating to a damaging or cytotoxic compartment (Simeoni et al., 2004). Conversely, the Signal Distribution model emphasizes that the drug does not cause cellular damage; rather, signaling cascades lead to cytotoxicity (Lobo and Balthasar, 2002). Monica Simeoni’s TGI model uses both the Log Kill Hypothesis and the Cell Distribution model to formulate a set of differential equations that capture drug efficacy as a measure of cell proliferation relative to the rate of drug-induced damage.

## PK-PD tumor growth inhibition models

### Simeoni–tumor growth kinetics

In 2004, Simeoni and colleagues developed a PK-PD model to fit the drug response of mouse xenograft studies (Simeoni et al., 2004; Rocchetti et al., 2007). While this is an older model than Sorger’s GRI proposal, it addresses key gaps in traditional pharmacology (Hafner et al., 2016; Simeoni et al., 2004). It is thought that the dynamic nature of drug response can be used to help understand the clinical translation gap by approximating the dose required for clinical efficacy. This model’s utility lies in its ability to combine drug concentration potency and tumor proliferation to understand clinical efficacy (Figure 4A) (Simeoni et al., 2004; Rocchetti et al., 2007). The original purpose of this model was to better predict the active dose required in human patients and to bridge the gap between animal studies and human clinical trials (Rocchetti et al., 2007). We hypothesize that certain pharmacodynamic features emphasized in Simeoni’s TGI model, including delayed cellular responses to drug exposure, can be examined in controlled *in vitro* systems, while acknowledging that key *in vivo* determinants such as drug distribution are not captured.

Simeoni highlights four key steps in drug response: the first is that the drug must damage the cells, followed by downstream killing (Figure 4B). The downstream killing is divided into three steps to account for the observed lag in the response, similar to the downstream effects following initial target binding. While only using three killing phases or steps is an oversimplification of drug response due to variations in mechanisms of action across drug classes, this phenomenological term can be applied to multiple oncology drugs to recapitulate drug response. The model captures the mechanism-inspired structures of drug efficacy using three parameters: the rate of proliferation (*k*_*PRO*_), the rate of drug damage (*k*_*DMG*_), and the rate of cell killing (*k*_*KILL*_). The response dynamics within the Simeoni model are not generally identifiable as distinct biological rate constants; by contrast, Sorger’s k_DRUG_ represents an overall phenomenological parameter that aggregates these contributions into a single term (Hafner et al., 2016). The key insight from this model is that a minimum time is required to elicit the drug’s desired response, as captured by the multistep process. Additionally, the timing of the peak is determined by the competition between cell proliferation and the drug’s efficacy. We can use the ratio of *k*_*PRO*_ to *k*_*DMG*_ to approximate a model-dependent concentration threshold, C_t_, which is the theoretical steady-state concentration required to induce tumor shrinkage under the model’s assumptions (Figure 4A, equation) (Simeoni et al., 2004; Rocchetti et al., 2007). This threshold may help inform the selection of experimentally relevant concentration ranges for animal studies, and, by extension, guide dose-selection considerations for subsequent clinical trials. Many oncology drugs were historically designed to target cell-cycle–associated processes because malignant cells were recognized to divide more rapidly than normal tissues (Knox et al., 2024). It follows that the faster the cell cycle, the quicker or more efficacious the response; however, for a drug to induce tumor shrinkage, the drug’s ability to induce cellular damage must be faster than cancer’s proliferation.

### Koch

In 2009, Gilbert Koch and colleagues developed an adaptation of the Simeoni TGI model, a deviation from a mechanistic model, to aid in drug efficacy studies of more complex drug responses, such as those in combination therapy (Koch et al., 2009). One key difference, as mentioned earlier, is that Simeoni’s model assumes an automatic switch from exponential to linear growth once a threshold tumor volume is reached. In contrast, Koch proposes a gradual slowing of tumor growth. Additionally, the time required for cytotoxic chemotherapy to induce cell death (τ) is explicitly defined (Koch et al., 2009). This is important for developing and potentially refining dosing regimens for chemotherapies to ensure that the drug exposure time is longer than the minimum time required to induce tumor death. To account for the timing of drug exposure, a new formula for the Concentration Threshold was developed, along with a method to evaluate interactions among multiple therapies in combination treatment (Koch et al., 2009).

### PK-PD TGI model advantages, strategies, and challenges

The practical challenge of these PK-PD TGI models is that they require multiple time points to resolve whether there is a transition in growth kinetics associated with the minimum efficacy time. While Sorger GRI model is an easy method to implement using the equation outlined in Figure 2B, it measures only two time points, the endpoint and the initial time point, making it impossible to resolve when or if there is a transition from proliferation to cytostatic or cytotoxic patterns that can sometimes be observed in modern PK-PD models. This makes it more challenging to identify the transition point at a single time point because the cell-killing process is a single-step, monotonic process. In drug-perturbed growth curves in both xenograft and cell-based assays using frequent or continuous monitoring, when tumor shrinkage is occurring, a peak is sometimes observed at the transition point from proliferation to cytostatic/cytotoxic effects (Simeoni et al., 2004; Rocchetti et al., 2007). While not defined in Koch’s model, the time required for the drug to enter the tissue of interest and/or the cell, in addition to target binding signaling cascades, causes another potential source for a lag in the drug’s ability to induce cytotoxicity in the tumor (Meibohm and Derenforf, 1997; Rizk et al., 2017). While the use of multiple time points in cell-based methods is more costly and intensive, capturing the pharmacokinetic-pharmacodynamic response could prove to be beneficial for increasing the translational potential *of in vitro* assays by reducing false positives, but again is insufficient by itself to improve clinical translation. Continuous monitoring assays using either fluorescent microscopy or plate-based assays provide an opportunity to monitor drug kinetics at various concentrations to observe changes in cell growth following exposure (Duellman et al., 2015; Polanco et al., 2022; Gross et al., 2023). While continuous drug monitoring allows an improved understanding of drug kinetics in cancer cells *in vitro*, another limitation is that many of these *in vitro* methods are exposed to a steady-state concentration throughout the experiment. Additionally, because of the heterogeneity of many cancers, the proliferation term would be an aggregate across both sensitive and resistant cell populations, or may not capture senescent cells. This highlights the limitations of immortalized cell lines and the need for improved cell-based models that are able to capture the variability as well as the influence of the tumor microenvironment on disease progression. While there are many areas for improvement and limited research on applying PK-PD principles *in vitro*, we believe monitoring drug kinetics in more clinically relevant models could aid in understanding drug response in greater depth.

## Novel treatment options for oncology

As mentioned earlier, many *in vitro* and preclinical studies have historically focused on the cancer’s response to cytotoxic chemotherapies, but as the field has evolved, we have gained a better understanding of how both tumor heterogeneity and the tumor microenvironment contribute to disease progression and drug response (Morton et al., 2011; Fallahi-Sichani et al., 2013; Letai et al., 2022; Son et al., 2017; Wang et al., 2023). While small-molecule chemotherapies are the standard of care in many cancer treatment regimens, novel biological therapies, particularly antibodies targeting programmed cell death 1 (PD-1) and programmed cell death ligand-1 (PDL-1), have become increasingly indicated on top of classical regimens (Fundytus et al., 2021; Haq et al., 2024). Another advance in oncology drug discovery is the development of cancer vaccines, including mRNA and viral vector approaches (Zaidi et al., 2025; Vergati et al., 2010). The most successful of the cancer vaccines have been preventative methods for oncogenic viruses such as human papillomavirus or hepatitis B, but more recently, the approval of sipuleucel-T for refractory prostate cancer. In addition, the advances in mRNA technology have opened the door to improvements in cancer vaccine treatments that modulate the immune environment (Zaidi et al., 2025; Vergati et al., 2010). Another area of recent advancement in oncology treatments is the development of chimeric antigen receptor T cells (CAR-T), which modify a patient’s T cells to identify and kill tumor cells (Rodgers et al., 2016). In addition to biologics that target multiple cell types, many new small molecules have multiple targets through either protein-protein interactions or induce protein degradation, with a focus on using molecular interaction networks to rewire cellular processes (Fu et al., 2025; Douglass and Miller, 2025; Shu et al., 2025). Other groups focus on improving drug delivery and isolating off-target cytotoxicity through methods such as nanoparticles or antibody-drug conjugates (Gavas et al., 2021; Fu et al., 2022). As many of these drugs modulate responses within the tumor microenvironment or do not follow the traditional pharmacodynamic paradigm, this highlights the need to move toward more comprehensive efficacy models that incorporate multiple cell types and timepoints to better understand disease progression, rather than focusing solely on single-target protein binding.

## Advances in cell-culture methods

One of the most significant contributors to the high cost of drug development is the low success rate in clinical drug development, with even higher failure rates for cancer therapeutic agents (Khanna, 2012; Sun et al., 2022). Since 50% of drug failures in clinics are due to a lack of efficacy, this highlights the poor translational potential of drugs for both the development of novel therapeutics for underserved patient populations and *ex vivo* screening in precision oncology (Carrera et al., 2018; Sun et al., 2022). A large number of drugs perform well in preclinical models but show poor clinical efficacy, leading to “false positives” in drug discovery (Sun et al., 2022; Seyhan, 2019). Studies have shown that more complex cell methods, such as 3D culture, can help reduce the rate of false positives by 10%–40% (Ganesh et al., 2019; Guillen et al., 2022; Narasimhan et al., 2020; Wensink et al., 2021). Similarly, *ex vivo* studies show a substantial reduction in the rate of false positives, yet twice as many drugs exhibit higher sensitivity in cell-based cytotoxicity assays, as measured by metrics such as IC_50_ or AUC, compared to those observed in the clinic (Kurtz et al., 2017; Von Hoff, 1990). However, these advanced cell culture methods do not often consider time-response or tissue-exposure metrics that are critical for *in vivo* translation (Hafner et al., 2016). Many groups are working to develop improved co-culture and organoid methods to recapitulate patient tumors and address factors beyond tumor heterogeneity, such as the tumor microenvironment (Wu and Swartz, 2014; Sánchez-de-Diego et al., 2025; Tan et al., 2021; Shukla et al., 2022; Moshksayan et al., 2025; Gabriel et al., 2022; Albritton and Miller, 2017). In addition to modulating the tumor microenvironment, tools such as organ-on-a-chip technology and microfluidics are able to recapitulate physiological systems (Gabriel et al., 2022; Moshksayan et al., 2025; Sánchez-de-Diego et al., 2025). These tools allow the simulation of drug distribution and immune circulation. As the oncology field moves towards more advanced *ex vivo* screening, the need for better metrics to quantify drug translation is vital. By decreasing false-positive rates with innovative time-based metrics, we can further improve drug translation when these methods are applied to improved cellular models. Many labs are adapting innovative time-based drug screening through either fluorescent microscopy or plate-based assays (Hsieh et al., 2017; Evans et al., 2019; Polanco et al., 2022; Gross et al., 2023; Duellman et al., 2015). These innovations in cellular models, in addition to time-based experiments, have the potential to narrow the gap between the laboratory and the clinic, thereby improving bench-to-bedside translation.

## Conclusion

The 5-year survival rate across all cancer types has increased from 49% in the 1970s to 69% in the years 2014–2020, but while some cancers have remarkably high survival rates, others, such as pancreatic cancer, have survival rates as low as 13% (Siegel et al., 2025; Sherer et al., 2022; Khwaja et al., 2016). Early detection and innovative treatment methods have improved patient outcomes, but for some cancer types, there remain significant gaps in effective treatments (Khwaja et al., 2016; Siegel et al., 2025). Additionally, while some patients respond to the standard of care, there are still considerable variations in patient response, especially within the Native American and Black populations in the US (Siegel et al., 2025, 202; Sherer et al., 2022; Arias et al., 2021; Bach, 2002). While research is ongoing to raise survival rates across hard-to-treat cancer types, one of the contributing factors to poor patient prognosis is “Financial Toxicity” (Carrera et al., 2018; Experts in Chronic Myeloid Leukemia, 2013; Abu-Khalaf et al., 2019). Oncology drugs, especially novel treatments such as biologics, while showing improved efficacy in some cases, are particularly expensive due to the high failure rate in drug development, particularly in the “valley of death” (Carrera et al., 2018; Abu-Khalaf et al., 2019). A better understanding of the limitations of our conceptual frameworks in drug pharmacology is vital to begin bridging the gap. This review aims to highlight recent advances in cancer pharmacology and limitations within the field that might contribute to the translational gap.

Several factors affect the efficacy of chemotherapies, including not only their pharmacodynamic effect but also their pharmacokinetic parameters (Figures 3, 4). Traditionally, preclinical work, particularly *in vitro*, focuses on optimizing drug binding and pharmacodynamics, while clinical studies focus on integrating both the pharmacokinetic and pharmacodynamic responses to drug treatments. We believe this highlights a gap in conceptual frameworks that contributes to the translational gap. To address this, many labs have begun to incorporate time and/or phenotypic responses into their analyses of drug efficacy in preclinical models, using methods such as Sorger’s GRI model, thereby beginning to bridge the translation gap. While a shift in preclinical models to incorporate PK-PD principles is vital to reduce false positives, it is not the only thing needed to improve clinical translation. In addition, the highly heterogeneous tumor environment, both within the tumor and in the surrounding microenvironment, influences drug response, which can be exacerbated by the patient’s genetic background. There is a need to consider drug discovery and drug translation as a continuous feedback loop between pharmacodynamics and pharmacokinetics. The use of feedback loop implementations, improved *in vitro* experimental, and pharmacokinetic-pharmacodynamic principles could help increase translation potential (Chien et al., 2005). In recent years, numerous developments in tools for creating more biologically relevant systems have emerged, including continuous cell monitoring, co-culture systems, microfluidics, and patient-derived organoids. In addition to advances within *in vitro* methods, drug radiotracing has enabled clinicians and scientists to understand drug distribution through less invasive methods, which can be used to improve drug localization within the diseased tissue and tumor.

In addition to a shift in conceptual frameworks, there is a need for more robust database infrastructures to support both medicinal chemists and translational scientists in evaluating the factors that impact drug efficacy beyond traditional target binding and drug clearance. Databases such as CCLE have proven instrumental in aiding our understanding of gene expression and drug sensitivity (Barretina et al., 2012). Databases on drug distribution and xenograft studies could help elucidate variation in drug response, complementing genetic data, since most pharmacokinetic data is considered inaccessible. We hypothesize that by developing more comprehensive drug pharmacokinetic databases and incorporating PK-PD modeling into the earliest stages of drug testing in improved cellular models, we can help bridge the translational gap in cancer pharmacology, ultimately leading to improved treatments for patients.

## Supplementary material

SUPPLEMENTARY FIGURE 1 Comparison of *in vitro* Growth Rate Assay and Clinical Growth Rate (RECIST). A Visual Representation of the Comparison of Response *in vitro *versus in human clinical patients prior or post-treatment. CR is a complete responder, PR is partial responder, SD is stable disease, and PD is progressive disease.
