# Supplementary material for: Gaps and paths forward in cancer pharmacology and translational research
Source: Front Pharmacol. 2026 Apr 10;17:1779049. doi: 10.3389/fphar.2026.1779049 (PMC13106357; doi:10.3389/fphar.2026.1779049)
Supplement: Supplementary file 1 [file Supplementaryfile1.docx]

| **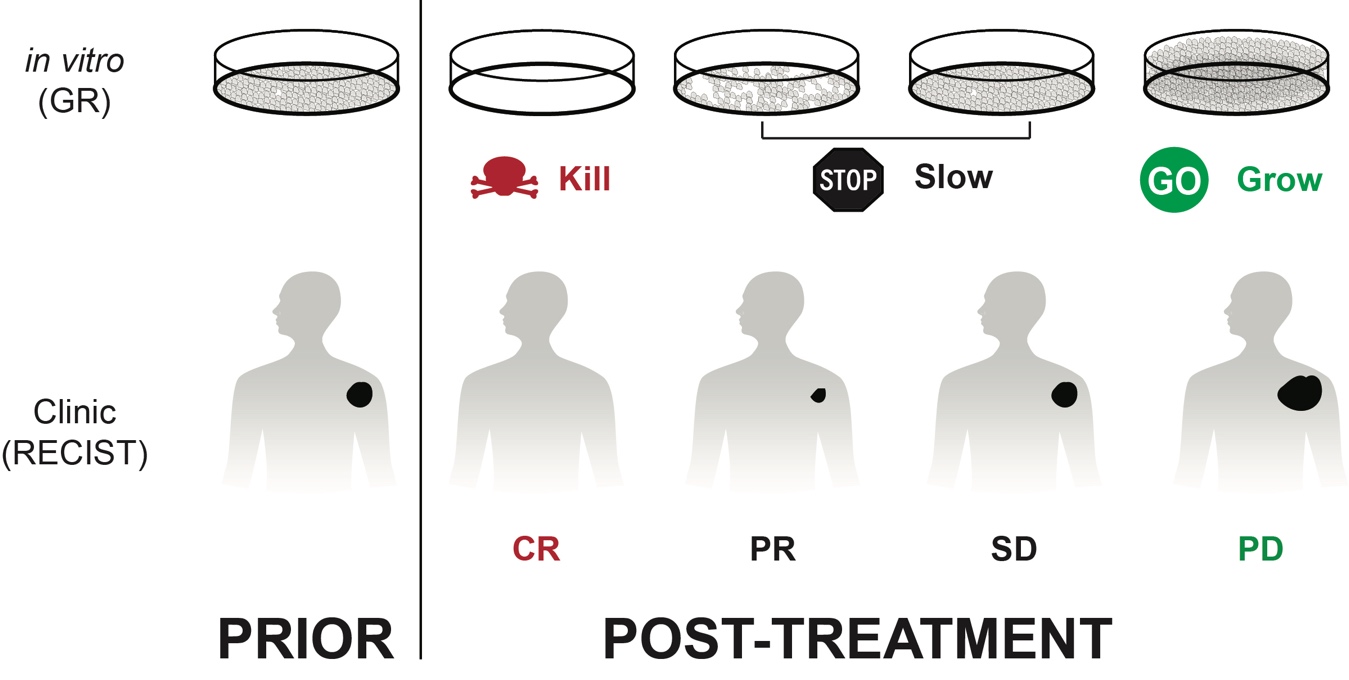** |
| --- |
| **Supplemental Figure 1 – Comparison of *in vitro* Growth Rate Assay and Clinical Growth Rate (RECIST).** A Visual Representation of the Comparison of Response *in vitro* versus in human clinical patients prior or post-treatment. CR is a complete responder, PR is partial responder, SD is stable disease, and PD is progressive disease. |
